# The dual nature of trehalose in citrus canker disease: a virulence factor for *Xanthomonas citri* subsp. *citri* and a trigger for plant defence responses

**DOI:** 10.1093/jxb/erv095

**Published:** 2015-03-14

**Authors:** Ainelén Piazza, Tamara Zimaro, Betiana S. Garavaglia, Florencia A. Ficarra, Ludivine Thomas, Claudius Marondedze, Regina Feil, John E. Lunn, Chris Gehring, Jorgelina Ottado, Natalia Gottig

**Affiliations:** ^1^Instituto de Biología Molecular y Celular de Rosario, Consejo Nacional de Investigaciones Científicas y Técnicas (IBR-CONICET) and Facultad de Ciencias Bioquímicas y Farmacéuticas, Universidad Nacional de Rosario, Ocampo y Esmeralda, Rosario 2000, Argentina; ^2^Biological and Environmental Science and Engineering Division, King Abdullah University of Science and Technology, Thuwal 23955-6900, Saudi Arabia; ^3^Max Planck Institute of Molecular Plant Physiology, Wissenschaftspark Golm, Am Mühlenberg 1, 14476 Potsdam (OT) Golm, Germany

**Keywords:** Canker, citrus, defence, *otsA*, trehalose, *Xanthomonas*.

## Abstract

*Xanthomonas citri* subsp. *citri* (Xcc) is a bacterial pathogen that causes citrus canker in susceptible *Citrus* spp. The Xcc genome contains genes encoding enzymes from three separate pathways of trehalose biosynthesis. Expression of genes encoding trehalose-6-phosphate synthase (*otsA*) and trehalose phosphatase (*otsB*) was highly induced during canker development, suggesting that the two-step pathway of trehalose biosynthesis via trehalose-6-phosphate has a function in pathogenesis. This pathway was eliminated from the bacterium by deletion of the *otsA* gene. The resulting XccΔotsA mutant produced less trehalose than the wild-type strain, was less resistant to salt and oxidative stresses, and was less able to colonize plant tissues. Gene expression and proteomic analyses of infected leaves showed that infection with XccΔotsA triggered only weak defence responses in the plant compared with infection with Xcc, and had less impact on the host plant’s metabolism than the wild-type strain. These results suggested that trehalose of bacterial origin, synthesized via the otsA–otsB pathway, in Xcc, plays a role in modifying the host plant’s metabolism to its own advantage but is also perceived by the plant as a sign of pathogen attack. Thus, trehalose biosynthesis has both positive and negative consequences for Xcc. On the one hand, it enables this bacterial pathogen to survive in the inhospitable environment of the leaf surface before infection and exploit the host plant’s resources after infection, but on the other hand, it is a tell-tale sign of the pathogen’s presence that triggers the plant to defend itself against infection.

## Highlight

Trehalose is a double-edged sword for both partners in the citrus–*Xanthomonas* interaction, as it is necessary for bacterial survival but also triggers citrus defence responses.

## Introduction

Trehalose is a non-reducing disaccharide in which two glucose molecules are joined together by an α,α-(1-1) glycosidic bond. This disaccharide occurs in many organisms, such as bacteria, fungi, and invertebrates, which use it for carbohydrate storage and as a compatible solute in protection against diverse stresses (Miller and Wood, 1996; Benaroudj *et al.*, 2001; Elbein *et al.*, 2003; Makihara *et al.*, 2005). It is thought that this protective role is due to the property of trehalose to stabilize both proteins and membranes under stress conditions (Iturriaga *et al.*, 2009). Many non-vascular plants and a small number of desiccation-tolerant resurrection plants accumulate trehalose, but levels are generally very low in most angiosperms, and hence unlikely to make a significant direct contribution to osmoregulation or carbohydrate reserves (Iturriaga *et al.*, 2009). For this reason, it was for a long time assumed that this disaccharide was unimportant in most flowering plants. However, in recent years, it has been demonstrated that trehalose and its precursor trehalose-6-phosphate (T6P) are important signal molecules that affect many metabolic pathways, developmental processes, and stress responses in plants (Lunn *et al.*, 2014).

In symbiotic interactions between legumes and rhizobial bacteria, trehalose synthesis increases during drought, potentially protecting both the host and symbiont from stress (Farias-Rodriguez *et al.*, 1998). Maize plants inoculated with *Azospirillum brasilense* that overproduced trehalose were more resistant to drought stress than plants inoculated with the wild-type (WT) bacteria (Rodriguez-Salazar *et al.*, 2009). Further evidence that trehalose has a protective function in rhizobial symbioses came from overexpression of T6P synthase (TPS) in *Rhizobium etli*, which improved drought tolerance (Suarez *et al.*, 2008), while blocking trehalose catabolism increased nodulation efficiency of *Synorhizobium* spp. on alfalfa (*Medicago sativa*) and barrel medic (*Medicago truncatula*) (Ampomah *et al.*, 2008), and of *Bradyrhizobium japonicum* on soybean (*Glycine max*) (Sugawara *et al.*, 2010). In the ectomycorrhizal fungus *Amanita muscaria*, fungal genes encoding enzymes of trehalose metabolism were induced during mycorrhizal symbiosis (Lopez *et al.*, 2007), while in the interaction of *Pisolithus microcarpus* with *Eucalyptus globulus*, mycorrhizal colonization increased the allocation of carbon to trehalose in the mycelium (Martin *et al.*, 1998).

A few studies have suggested a role for trehalose metabolism in plant interactions with microbial pathogens (Fernandez *et al.*, 2010). The deletion of the *TPS* gene in the fungus *Magnaporthe oryzae*, the causal agent of rice blast disease, abolishes the ability of the fungus to synthesize trehalose and weakens its pathogenicity, either by interfering with the establishment of high turgor in the appressorium or with subsequent hyphal penetration (Foster *et al.*, 2003). In *Arabidopsis thaliana* plants infected with the rhizarian *Plasmodiophora brassicae*, the causal agent of clubroot disease, trehalose accumulates in infected organs due to the upregulation of a *Plasmodiophora brassicae TPS* gene (Brodmann *et al.*, 2002). In this interaction, trehalose appeared to interfere with plant carbon metabolism in a way that favoured pathogen development. Infection with *Plasmodiophora brassicae* induces expression of the host *TREHALASE* gene, and this has been suggested to be a defence mechanism preventing excess accumulation of trehalose in the plant cells, which might otherwise perturb the plant metabolism (Brodmann *et al.*, 2002). This hypothesis was supported by a finding that partial resistance to clubroot infection in *Arabidopsis thaliana* is linked to higher tolerance to trehalose (Gravot *et al.*, 2011). *Pseudomonas syringae* pv. *tomato* is a bacterial pathogen of tomato. Mutant strains of the bacterium that do not accumulate trehalose had lower rates of survival on tomato leaves and also on leaves of soybean, a non-host plant, suggesting that trehalose is a significant factor in conferring tolerance to abiotic stress found in the phylloplane (Freeman *et al.*, 2010). Another recent study showed that synthesis of trehalose by *Pseudomonas aeruginosa* strain PA14 is necessary for pathogen growth in the intercellular spaces of *Arabidopsis thaliana* leaves but not when this broad-range pathogen infects non-plant hosts (Djonovic *et al.*, 2013).

It has been demonstrated that exogenous trehalose acts as an elicitor of plant defence mechanisms, with microarray analysis of *Arabidopsis thaliana* grown on trehalose-containing medium showing the induction of multiple genes involved in plant defence against pathogens (Aghdasi *et al.*, 2008; Bae *et al.*, 2005a, b). Furthermore, exogenous application of trehalose to wheat plants can induce resistance to *Blumeria graminis*, a necrotrophic fungus that causes powdery mildew (Reignault *et al.*, 2001; Renard-Merlier *et al.*, 2007; Tayeh *et al.*, 2014). In contrast, exogenous application of trehalose to tomato plants did not confer resistance to powdery mildew (*Oidium* spp.), although treatment with validamycin A, an inhibitor of trehalase, did induce resistance to *Fusarium* wilt (*Fusarium oxysporum* f. sp. *lycopersici*) and tomato late blight (*Phytophthora infestans*) (Ishikawa *et al.*, 2005).

In order to shed new light on the role of this disaccharide in plant–pathogen interactions, we studied the possible role of trehalose in the interaction of *Xanthomonas citri* subsp. *citri* (Xcc) with citrus plants. Xcc is a Gram-negative plant-pathogenic bacterium that causes canker on *Citrus* spp. This phytopathogen invades host plant tissues through either the stomata or wounds, and then colonizes the apoplast of fruits, foliage, and young stems, with symptoms of infection appearing as raised corky lesions known as cankers. At the final stage, the plant epidermal tissue is broken down due to cell hyperplasia, which allows dispersal of the bacteria to other plants by wind or rain. Persistent and severe disease can lead to defoliation, dieback, and fruit drop, thereby causing serious economic losses for commercial growers (Graham *et al.*, 2004). A mutant strain of Xcc (XccΔotsA) was generated that lacks the TPS-encoding *otsA* gene, eliminating its capacity to synthesize trehalose via T6P. The ability of the Xcc∆otsA mutant to resist environmental stress conditions and to colonize plant tissues was compared with the Xcc WT strain (XccWT) and we determined that trehalose is a significant factor in the infection process leading to citrus canker disease.

## Material and methods

### Bacterial strains, culture conditions and media

Xcc strain Xcc99-1330 was isolated from *Citrus sinensis* (INTA Bella Vista, Argentina). The Xcc∆otsA mutant was obtained by marker-exchange mutagenesis; the flanking regions of XAC3211 (Xcc *otsA* gene) were amplified by PCR with the following pairs of oligonucleotides: 3′otsA-LX: 5′-ATCA*CTCGAG*CGCAAGAACAACATCAACCA-3′ and 3′otsA-RH: 5′-ATAC*AAGCTT*ATCGGCCAGCTCGACATT-3′; and 5′otsA-LH: 5′-ATCA*AAGCTT*GGCAATGACCACCTCGATT-3′ and 5′otsA-RS: 5′-TCC*CCCGGG*AACAAGCCAACCGCCAAG-3′. Underlined are the restriction sites for *Xho*I, *Hin*dIII and *Sma*I, respectively. Amplified products were cloned sequentially in pK19mobGII (Katzen *et al.*, 1999) previously digested with *Xho*I and *Hin*dIII and then with *Hin*dIII and *Sal*I. The resulting plasmid was digested with *Hin*dIII and in this site the 2.3 kbp *aacC1* cassette obtained from pWKR329B coding for gentamicin resistance (Reece and Phillips, 1995) was subcloned. As described previously, *Escherichia coli* S17-1 cells transformed with this vector were conjugated to Xcc and selected for gentamicin resistance and kanamycin sensitivity to obtain the Xcc∆otsA mutant (Gottig *et al.*, 2008). The Xcc∆otsAc complemented strain was constructed by cloning the gene *otsA* in the replicative plasmid pBBR1MCS-5 (Kovach *et al.*, 1995) under the control of the *lacZ* promoter. This region was amplified from Xcc genomic DNA with the following oligonucleotides: otsA-LE (5′-AGTCGAATTCGATGAGTCGTTTGGTGGTGGC-3′) and otsA-RH (5′-TGCTAAGCTTCTACACCTCAGAGAGCGCT-3′) and cloned into pBBR1MCS-5 previously digested with the restriction enzymes *Eco*RI and *Hin*dIII. The resulting construction was electroporated into the Xcc∆otsA strain, and the complemented mutant strain was selected by kanamycin resistance. All strains were grown at 28 °C in SB, NB (Dunger *et al.*, 2007), or in XVM2 medium (Gottig *et al.*, 2008). Antibiotics were used at the following final concentrations: 25 μg ml^–1^ of ampicillin, 5 μg ml^–1^ of gentamicin, and 40 μg ml^–1^ of kanamycin.

### RNA preparation and quantitative reverse transcription-PCR (RT-qPCR)

Total RNA from bacterial cultures grown at the indicated conditions, from bacteria recovered from *C. sinensis* leaves infected with the bacterial strains, from infected leaves or from leaves treated with trehalose, maltose, and sucrose were isolated using TRIzol^®^ reagent (Invitrogen), according to the manufacturer’s instructions. RT-qPCRs were performed as described previously (Sgro *et al.*, 2012), with the specific oligonucleotides detailed in Supplementary Table S2 at *JXB* online. Values are the means of four biological replicates with three technical replicates each.

### Quantification of trehalose in XVM2 medium

The strains were grown for 16h in XVM2 medium and trehalose was extracted from bacterial cells and quantified by enzymatic reactions as described previously (Sugawara *et al.*, 2010). Protein concentrations in bacterial extracts were measured according to Bradford (1976), and the results were calculated as nmol of trehalose per total protein. For each strain, four flasks with 10ml of bacterial culture were used, and the experiment was repeated three times with similar results.

### Salt and oxidative stress survival assays

The strains were grown for 16h in flasks with 10ml of SB medium supplemented with the appropriate antibiotics, with shaking at 28 °C. A 1:50 dilution of these cultures was performed in XVM2 medium without NaCl and with 300mM NaCl. These cultures were grown for 16h with shaking at 28 °C and the cell density [colony-forming units (cfu) ml^–1^] was quantified by inoculating serial dilutions on SB medium solidified with 1.5% agar. The resistance to H_2_O_2_ was evaluated as described previously (Tondo *et al.*, 2010). The percentage of surviving cells was calculated from the ratio of cfu ml^–1^ obtained in each treatment and the cfu ml^–1^ obtained without treatment. In each experiment, a duplicate for each strain was performed and this was repeated three times.

### Plant material and inoculations

*C. sinensis* cv. Valencia plants used for Xcc infection assays were grown in a growth chamber in incandescent light at 28 °C with a photoperiod of 16h. Bacteria were cultured in SB broth to an optical density at 600nm (OD_600_) of 1, harvested by centrifugation at 3500*g*, and resuspended in 10mM MgCl_2_ at 10^3^, 10^7^, or 10^9^ cfu ml^–1^. Leaf-associated growth was evaluated as described previously (Dunger *et al.*, 2007). To mimic the natural infection process, plants were inoculated by spraying bacterial suspensions (10^9^ cfu ml^–1^) on citrus leaves. Cankers were counted from 20 citrus leaves inoculated with the different strains, the areas of the leaves were measured from digitalized images using Adobe Photoshop software, and the results are presented as cankers cm^–2^ of leaf tissue. For the evaluation of disease symptoms by infiltration, when bacteria were infiltrated at 10^3^ cfu ml^–1^, cankers were counted from 15 infiltrated citrus leaves per Xcc strain as above. Following infiltration of the strains at 10^7^ cfu ml^–1^, growth assays at the indicated times and quantification of the necrotic areas were performed as described previously (Gottig *et al.*, 2008).

### Quantification of exopolysaccharide (EPS) production

Quantification of EPS production was performed as described previously (Dunger *et al.*, 2007). Results were expressed in g of EPS per litre of culture. Quadruplicate measurements were made for each strain and an average of all measurements was obtained.

### Quantification of trehalose *in planta*

Trehalose was also measured from five leaves infiltrated with XccWT, Xcc∆otsA, or Xcc∆otsAc at 10^7^ cfu ml^–1^, or with 10mM MgCl_2_ as a mock-inoculated control at 3 and 6 d post-inoculation (dpi). Trehalose was extracted from frozen plant tissue using chloroform/methanol as described previously (Lunn *et al.*, 2006). The trehalose content of plant extracts was determined enzymatically with fluorometric detection as described by Carillo *et al.* (2013).

### Quantification of sugar phosphates and metabolites *in planta*

Chloroform extracts were prepared from five leaves infiltrated with XccWT, Xcc∆otsA, or Xcc∆otsAc (all at 10^7^ cfu ml^–1^), or with 10mM MgCl_2_ as a mock-inoculated control, and harvested at 3 and 6 dpi. T6P, α-maltose 1-phosphate (α-M1P), and other metabolic intermediates were measured by high-performance anion-exchange liquid chromatography coupled to tandem mass spectrometry (LC-MS/MS) as described previously (Lunn *et al.*, 2006).

### Proteomic analyses

Total plant proteins from leaves infiltrated with the XccWT and Xcc∆otsA strains and infiltrated with MgCl_2_ as a control were extracted at 3 dpi and labelled using a fluorescent cyanine three-dye strategy (CyDyes; GE Healthcare), as described previously (Garavaglia *et al.*, 2010). Proteins from leaves infiltrated with XccWT, Xcc∆otsA, and MgCl_2_ were labelled with Cy3, Cy5, and Cy2, respectively, according to the manufacturer’s instructions. Protein extractions were performed from three independent biological samples, and two technical replicate gels for each experiment were run. Protein separation, quantification by two-dimensional difference in-gel electrophoresis, comparative analysis, and protein identification were also carried out as described previously (Zimaro *et al.*, 2013). Normalized expression profile data were used to statistically assess changes in protein spot expression.

### Quantification of chlorophyll fluorescence parameters

Chlorophyll fluorescence parameters were measured in five leaves infiltrated with XccWT, Xcc∆otsA, or Xcc∆otsAc at 10^7^ cfu ml^–1^, or with 10mM MgCl_2_ as a control, at each time point, as described previously (Garavaglia *et al.*, 2010).

### 3,3′-Diaminobenzidine (DAB) staining

To visualize H_2_O_2_ accumulation, leaves infiltrated with the different strains or with pure trehalose were stained with DAB (Sigma, St Louis, USA). The infiltrated leaves were cut and the petioles were submerged in a 0.1% (w/v) DAB solution and kept in the dark overnight. The leaves were cleared in ethanol and observed and photographed in an optical microscope. DAB intensity was calculated from the digital photographs by the number of brown pixels relative to the total number of pixels covering the plant material, using Photoshop CS3 software. Average DAB measurements were calculated from at least 25 photographs from three independent experiments.

### Determination of trehalase activity

The leaves were infiltrated with the different strains or with trehalose and trehalase activity was quantified as described previously (Brodmann *et al.*, 2002). The protein concentration in the extracts was determined according to Bradford (1976), and trehalase activity was calculated as μmol of glucose per mg of total protein min^–1^ in five leaves for each treatment.

### Analysis of XccWT growth in citrus leaves pre-infiltrated with trehalose

Ten citrus leaves were infiltrated with H_2_O and the XcchrpB^–^ strain as controls and with 0.025mg ml^–1^ of trehalose. After 16h, these leaves were infiltrated with XccWT suspension at 10^7^ cfu ml^–1^. Growth assays were performed by grinding 0.8cm diameter leaf discs in 1ml of 10mM MgCl_2_, followed by serial dilutions and plating onto SB agar plates. Colonies were counted after 48h of incubation at 28 °C, and the results are presented as log cfu cm^–2^ of leaf tissue.

### Statistical analysis

In all figures, bars are the mean of the data and error bars are the standard deviation. All data were analysed by one-way analysis of variance (ANOVA) or Student’s *t*-test, as indicated in the figure and table legends. For the proteomic analysis, differentially expressed protein spots were assessed using Student’s *t*-test with a critical value of *P*≤0.05 and the permutation-based method, to avoid biased results that may arise within replicate gels if spot quantities are not normally distributed. The adjusted Bonferroni correction was applied for the false discovery rate to control the proportion of false positives in the result set. Principal component analysis was performed to determine samples and spots that contributed most to the variance and their relatedness. Only protein spots with a minimum of 1.2-fold change and *P*<0.05 were considered to be significantly differentially expressed between infected leaves.

## Results

### Expression of Xcc trehalose biosynthetic pathways in XVM2 medium and *in planta*

**Fig. 1.**
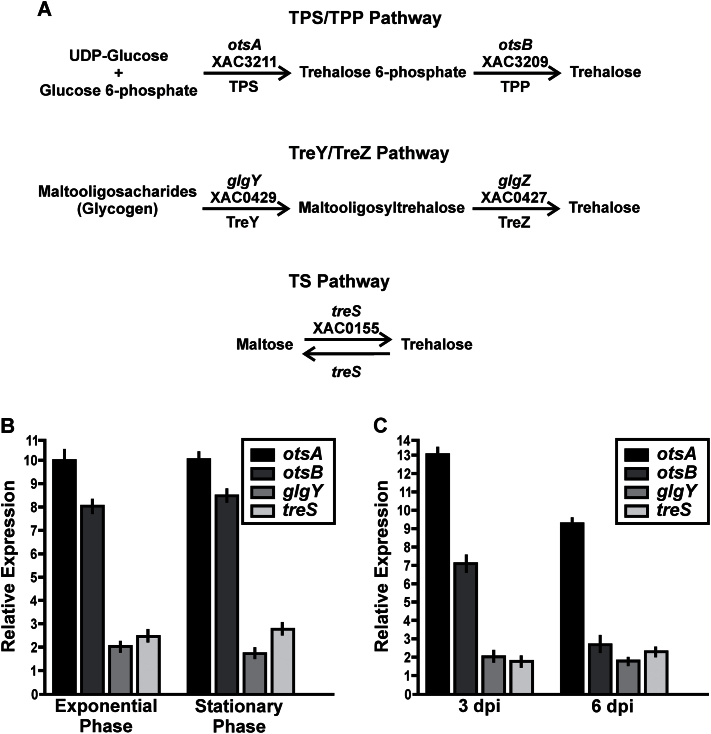
Analysis of the expression levels of *otsA*, *ostB*, *glgY*, and *TS* in XccWT by RT-qPCR assays. (A) Representation of trehalose biosynthetic pathways found in Xcc. The annotations of the enzymes in the Xcc genome are shown above the arrows. (B) RT-qPCR of XccWT RNA extracted from exponential-phase and stationary-phase cultures grown in XVM2. Bars indicate the expression levels of the genes at both growth phases in XVM2 medium relative to the expression levels in NB rich medium. Values are the means of four biological replicates with three technical replicates each. Error bars indicate standard deviation. (C) RT-qPCR of XccWT RNA obtained from bacteria present in infected citrus leaves at 0, 3, and 6 dpi. Bars indicate the expression levels of the genes at 3 and 6 dpi relative to the expression levels at time 0. Values are the means of four biological replicates with three technical replicates each. Error bars indicate standard deviation. Results were analysed by Student *t*-test (*P*<0.05) and one-way ANOVA (*P*<0.05).

The fully sequenced and annotated Xcc genome (da Silva *et al.*, 2002) was screened for the presence of open reading frames with similarity to known trehalose biosynthetic genes. Genes encoding enzymes of three different trehalose biosynthetic pathways were found (Fig. 1A): (i) the TPS–trehalose phosphatase TPP (otsA–otsB) pathway; (ii) the treY–treZ pathway; and (iii) the treS pathway. The TPS–TPP pathway is widespread among prokaryotes, and is the only route of trehalose biosynthesis in eukaryotes; it is unique in having T6P as an intermediate. TPS (EC 2.4.1.15) is encoded by the *otsA* gene (locus XAC3211) and catalyses the transfer of glucose from UDP-glucose to glucose-6-phosphate, forming T6P, which is then dephosphorylated to release free trehalose by TPP, encoded by the *otsB* gene (Xcc locus XAC3209) (Cabib and Leloir, 1958). In the treY–treZ pathway, (1→4)-α-d-glucan 1-α-d-glucosylmutase (EC 5.4.99.15) changes the glycosidic bond in terminal maltosyl moieties of maltodextrins from an α-(1–4) to an α,α-(1-1) configuration, and then the resulting trehalosyl moiety is released as free trehalose by 4-α-d-[(1→4)-α-d-glucano]trehalose trehalohydrolase (EC 3.2.1.141) (Maruta *et al.*, 1996). The archetypal genes encoding these two enzymes in *Arthrobacter* sp. Q36 were termed *treY* and *treZ*, respectively (Maruta *et al.*, 1996), but their orthologues in Xcc have been annotated as *glgY* (locus XAC0429) and *glgZ* (locus XAC0427), respectively. In the third pathway, maltose is isomerized to trehalose by trehalose synthase (EC 5.4.99.16), which is encoded by the *treS* gene (locus XAC0155) (Nishimoto *et al.*, 1996).

To investigate the potential contribution of each pathway to bacterial trehalose biosynthesis during pathogenesis, the expression of *otsA*, *otsB*, *glgY*, and *treS* was evaluated by RT-qPCR in XccWT grown either in XVM2, a minimal medium that mimics the nutritional conditions of plant tissues (Wengelnik *et al.*, 1996), or in NB rich medium for comparison. Total RNA was extracted from exponential- and stationary-phase XccWT cultures and the RT-qPCR assay was performed with gene-specific primers. At both growth phases, expression of the *otsA* and *otsB* genes was 8–10 times higher (*P*<0.05) in XVM2-grown cells than in those on NB medium, whereas *glgY* and *treS* were only induced about 2- to 3-fold by growth in XVM2 medium (*P*<0.05) (Fig. 1B). The expression of these genes was also evaluated during interaction of the pathogen with one of its most important host plants, *C. sinensis* (sweet orange). Total RNA was obtained from XccWT recovered from infected *C. sinensis* leaves at 0, 3, and 6 dpi. RT-qPCR showed that both *otsA* and *otsB* were strongly induced after infection, with 13- and 7-fold higher levels of transcripts at 3 dpi compared with time 0, respectively (Fig. 1C). High expression of *otsA* was maintained at 6 dpi, while expression of *otsB* was only twice as high as at time 0. In contrast, there was only a 2-fold induction of *glgY* and *treS* at 3 or 6 dpi (Fig. 1C). Assuming that these changes in gene expression are translated into corresponding increases in enzyme activity, these results suggested that the TPS–TPP pathway of trehalose biosynthesis is strongly and preferentially induced upon infection, implying that this pathway has a role in pathogenesis.

### Effects of salt and oxidative stress on the Xcc∆otsA mutant

**Fig. 2.**
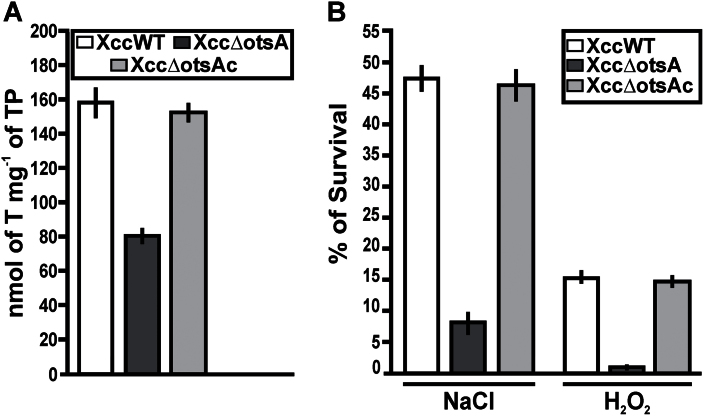
Quantification of trehalose and survival under salt and oxidative stress in the XccWT, Xcc∆otsA, and Xcc∆otsAc strains. (A) Trehalose content was quantified in the strains grown to the stationary phase in XVM2 medium. Bars indicate the nmol of trehalose (T) per mg of total protein (TP) of the bacterial pellets assayed. Values are the means of four cultures of each strain. Error bars indicate the standard deviation. (B) The cfu ml^–1^ of the strains in XVM2 medium without and in the presence of 300mM NaCl or 30mM H_2_O_2_ were calculated. Bars indicate the percentage survival under stress conditions relative to XVM2 medium. Values are the means of three independent experiments. Error bars indicate the standard deviation. The data were analysed for statistical differences by one-way ANOVA (*P*<0.05).

To investigate the role of the TPS–TPP pathway in Xcc pathogenicity, an *otsA* deletion mutant (Xcc∆otsA) was constructed by marker-exchange mutagenesis. Xcc∆otsA was complemented by conjugating plasmid pBBR1otsA, carrying a WT *otsA* gene, into this mutant, to give XccΔotsAc. When grown to stationary phase in XVM2 medium, the XccWT and complemented strains accumulated twice as much trehalose as the XccΔotsA mutant (Fig. 2A). The XccWT and complemented strains showed similarly high survival rates when grown in XVM2 medium supplemented with either 300mM NaCl (salt/osmotic stress) or 30mM H_2_O_2_ (oxidative stress), compared with growth in XVM2 medium alone. In contrast, survival rates of the XccΔotsA mutant were six to 15 times lower under these stress conditions (Fig. 2B).

### Pathogenicity of the Xcc∆otsA mutant in *C. sinensis* leaves

**Fig. 3.**
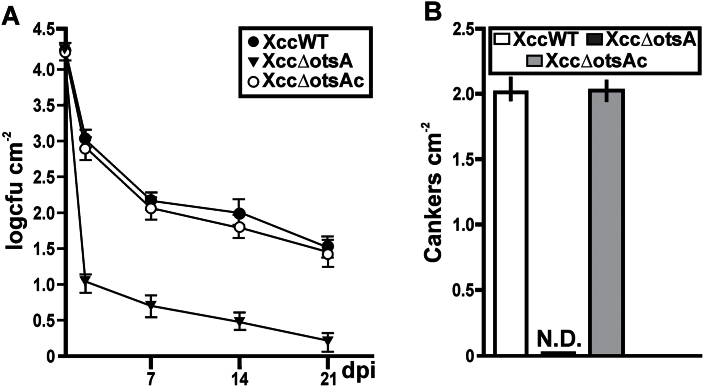
Analysis of the leaf-associated growth and the ability of canker formation of the XccWT, Xcc∆otsA, and Xcc∆otsAc strains using a natural method of infection. (A) Quantification of the population size of leaf-associated bacteria as log cfu cm^–2^ of citrus leaf surface on the indicated dpi. Values represent the mean of four leaves assayed for each strain. Error bars indicate the standard deviation. (B) Quantification of canker number on citrus leaves after 1 month of spray inoculation with the different strains at 10^9^ cfu ml^–1^. N.D., not detected. The data were analysed for statistical differences by one-way ANOVA (*P*<0.05).

The virulence of Xcc∆otsA was compared with the XccWT and complemented mutant strains on the host plant *C. sinensis* using different inoculation methods. The natural infection process was mimicked by spraying bacterial suspensions (10^9^ cfu ml^–1^) of the three strains on to the surface of separate leaves (Gottig *et al.*, 2009). Bacterial growth and canker numbers were monitored for up to 21 dpi. The population densities of XccWT and Xcc∆otsAc on the leaves decreased by over 95% within 2 dpi, and were less than 1% of the original density by 7 dpi, declining more slowly thereafter (Fig. 3A). The XccΔotsA mutant showed an even more precipitous decline after inoculation, falling to less than 0.1% of the original density at 2 dpi, and continuing to decrease until by 21 dpi the population density was 10 000 times lower than at inoculation (Fig. 3A). Thus, from 2 to 21 dpi, the population density of the XccΔotsA mutant was significantly different (*P*<0.05) from the WT and complemented strains, being one to two orders of magnitude lower at each time point. At 30 dpi, the XccWT and Xcc∆otsAc strains had produced about two cankers cm^–2^ of leaf area, but no cankers were observed on leaves inoculated with the Xcc∆otsA mutant (Fig. 3B).

**Fig. 4.**
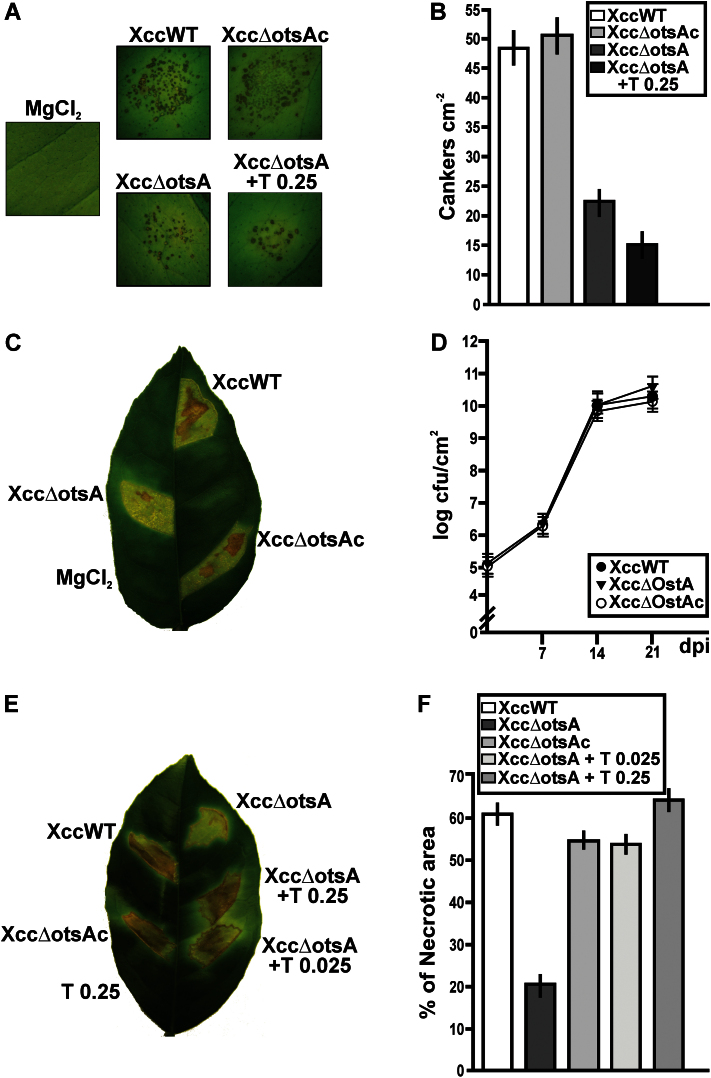
Analysis of the symptoms caused by XccWT, Xcc∆otsA, and Xcc∆otsAc in infiltrated citrus leaves. (A) The strains were inoculated at 10^3^ cfu ml^–1^ into the intercellular spaces of fully expanded leaves. The Xcc∆otsA mutant was also co-infiltrated with 0.25mg ml^–1^ of trehalose. Representative leaves of each infection are shown at 15 dpi. (B) Quantification of cankers cm^–2^ of infiltrated tissues of the leaves infiltrated as in (A). Bars are the means of 15 citrus leaves infiltrated with each strain. Error bars show the standard deviation. (C) Strains were inoculated at 10^7^ cfu ml^–1^ into the intercellular spaces of citrus leaves. A representative leaf is shown at 14 dpi. (D) Quantification of the population size of the strains inoculated in citrus leaves as described in (C). Values are the means of log cfu cm^–2^ of infiltrated tissues obtained from 15 infiltrated citrus leaves at different dpi. Error bars show the standard deviation. (E) A representative leaf of XccWT, Xcc∆otsAc, and Xcc∆otsA infections with co-infiltration of the Xcc∆otsA mutant with 0.025 and 0.25mg ml^–1^ of trehalose (T) at 14 dpi. (F) Quantification of the necrotic areas of the leaves infiltrated as in (C) and (E). Bars are the means of the percentages of necrotic areas for each strain from 15 infiltrated citrus leaves. Error bars show the standard deviation. The data were analysed for statistical differences by one-way ANOVA (*P*<0.05). (This figure is available in colour at *JXB* online.)

The infectivity of the three Xcc strains was also tested when the bacteria were pressure infiltrated directly into the leaves to bypass infection barriers at the leaf surface. Infiltration was performed with bacterial suspensions at two concentrations: 10^3^ and 10^7^ cfu ml^–1^, or with 10mM MgCl_2_ as a mock-inoculation control. In leaves infiltrated with the lower bacterial concentration, disease symptoms appeared as dispersed and isolated cankers (Fig. 4A), and at 15 dpi XccWT and Xcc∆otsAc formed about twice as many cankers cm^–2^ of leaf area as the Xcc∆otsA mutant (*P*<0.05) (Fig. 4B). Leaves were infiltrated with suspensions of the XccΔotsA mutant at 10^3^ cfu ml^–1^ containing either 0.025 or 0.25mg ml^–1^ of trehalose in the suspension medium, corresponding to about 100 and 1000 nmol g^–1^ of fresh weight of plant tissue, respectively. As described below, these concentrations coincide with the physiological range of trehalose measured in leaves infected with XccWT bacteria. Co-infiltrations of Xcc∆otsA with trehalose did not restore the number of cankers formed in the infections with XccWT, even at the highest trehalose concentration; in contrast, trehalose co-infiltrations impaired mutant canker formation (Fig. 4A, B). When the assay was performed with 0.025mg ml^–1^ of trehalose, the same results were observed (data not shown). When bacterial infiltrations were performed at 10^7^ cfu ml^–1^, all strains caused strong disease symptoms and no differences were observed at the onset of lesion formation or in lesion size between the strains. However, at 14 dpi, there was visibly less necrosis in the leaf sectors infected with the Xcc∆otsA mutant compared with those infected with XccWT or Xcc∆otsAc (Fig. 4C). Quantification of the necrotic areas confirmed that the differences were significant (*P*<0.05), with the XccWT and XccΔotsAc strains causing about three times more necrosis than XccΔotsA (Fig. 4F). XccWT, Xcc∆ostA, and Xcc∆ostAc bacterial growth was analysed on these infected citrus leaves and all strains grew similarly and achieved a population size of ~10^10^ cfu cm^–2^ at 14 dpi (Fig. 4D). Leaves were also infiltrated with suspensions of the XccΔotsA mutant containing either 0.025 or 0.25mg ml^–1^ of trehalose in the suspension medium. Both trehalose concentrations significantly increased the degree of necrosis compared with infiltration without trehalose (Fig. 4E), to levels comparable with the necrosis observed after infiltration with the XccWT and complemented strains (Fig. 4F). There were no visible lesions in leaf sectors infiltrated with 0.25mg ml^–1^ of trehalose alone (Fig. 4E). Co-infiltration of XccWT or Xcc∆otsAc with trehalose had no obvious effect on the severity of the necrosis (data not shown).

### Analyses of virulence-related factors in the Xcc∆otsA mutant

**Fig. 5.**
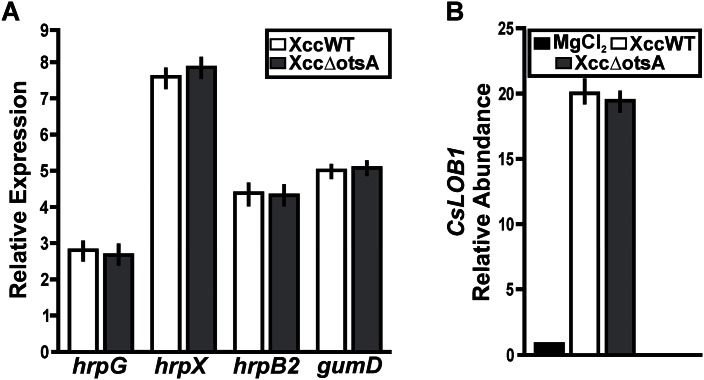
Analysis of Xcc∆otsA mutant virulence-related factors. (A) RT-qPCR of RNA obtained from XccWT and the Xcc∆otsA mutant present in infected citrus leaves at 0 and 3 dpi. Bars indicate the expression levels of the genes at 3 dpi relative to the expression levels at time 0. Values are the means of four biological replicates with three technical replicates each. Error bars show the standard deviation. (B) RT-qPCR to determine *CsLOB1* expression levels in leaves at 3 dpi with XccWT and the Xcc∆otsA mutant strain. Bars indicate the expression levels relative to buffer infiltrations. Values are the means of four biological replicates with three technical replicates each. Error bars show the standard deviation. The data were analysed for statistical differences by one-way ANOVA (*P*<0.05).

The weaker necrotic symptoms after infiltration with XccΔotsA (Fig. 4C–E) indicated that the mutant may be less virulent than the WT and complemented strains. To assess whether trehalose plays a direct or indirect role in determining the virulence of Xcc, the expression of virulence-related genes was compared in the mutant and WT control strains. RNA was extracted from XccWT and Xcc∆otsA cells recovered from *C. sinensis* leaves at 3 dpi with the respective strains, and the abundance of transcripts from the *hrpG*, *hrpX*, *hrpB2*, and *gumD* genes was determined by RT-qPCR. The *hrpB2* gene encodes a component of the type III secretion system (T3SS), which has two regulators that are encoded by the *hrpG* and *hrpX* genes, while *gumD* encodes undecaprenyl-phosphate glucose phosphotransferase (EC 2.7.8.31), an enzyme involved in EPS biosynthesis. At 3 dpi, transcript levels of all genes were 3- to 8-fold higher compared with time 0, but for each individual gene there was no significant difference (*P*>0.05) in transcript levels between XccWT and the Xcc∆otsA mutant (Fig. 5A). In addition, the EPS content of the XccΔotsA cells (3.30g l^–1^) was not significantly different from that of the XccWT cells (3.27g l^–1^). PthA4 is a transcriptional activator-like DNA-binding effector that is injected by Xcc into the host cell via the bacterial T3SS. There, it *trans*-activates expression of host plant genes, such as *CsLOB1*, which encodes a member of the LATERAL ORGAN BOUNDARIES family of transcription factors (Hu *et al.*, 2014; Pereira *et al.*, 2014). At 3 dpi, expression of *CsLOB1* was induced almost 20-fold by infection with XccWT compared with mock-inoculated control plants, and was similarly induced by infection with the XccΔotsA mutant (Fig. 5B). This suggested that similar amounts of the PthA4 virulence factor are expressed and secreted into the host plant cells by the two strains. Together, these results showed no evidence of differences between WT and mutant Xcc cells in the expression or activity of the three major virulence-related factors: the bacterial T3SS (*hrpG*, *hrpX*, and *hrpB2*), EPS biosynthesis (*gumD*), and PthA4.

### Effect of Xcc infection on metabolite levels in *C. sinensis* leaves

**Fig. 6.**
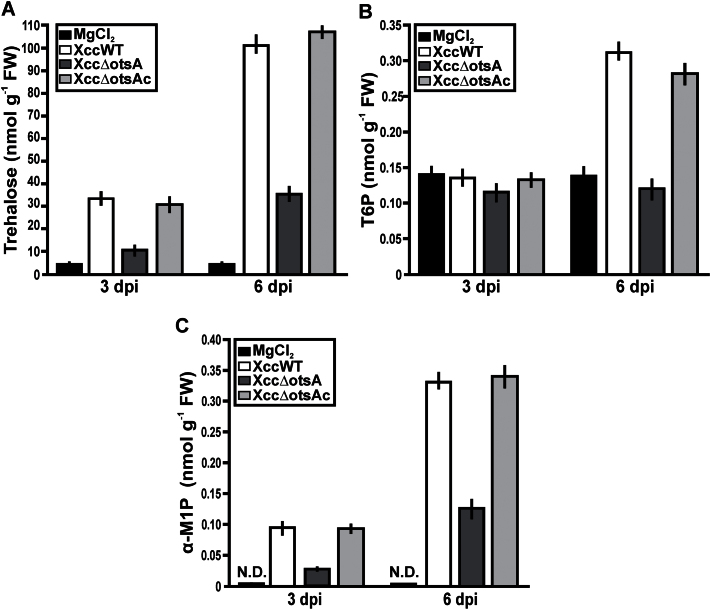
Quantification of trehalose, T6P, and α-M1P in leaves infected with XccWT, Xcc∆otsA, and Xcc∆otsAc strains. The leaves were infiltrated with the strains at 10^7^ cfu ml^–1^ and 10mM MgCl_2_, and after 3 and 6 dpi, quantification of trehalose (A), T6P (B), and α-M1P (C) as nmol g^–1^ of fresh weight (FW) of infiltrated tissue was performed. Bars are the means of the values of five infiltrated leaves with each strain at each time. Error bars show the standard error. N.D., not detected. Data were statically analysed by one-way ANOVA (*P*<0.05).

In addition to being a potential signal of pathogen attack in plants (Fernandez *et al.*, 2010), long-term exposure to exogenous trehalose can affect the level of T6P and other metabolites in plants (Brodmann *et al.*, 2002; Schluepmann *et al.*, 2004; Ma *et al.*, 2011), perturbing their growth and development (Schluepmann *et al.*, 2003; Wahl *et al.*, 2013). Trehalose levels were higher in Xcc-infected plants than in the mock-inoculated controls at 3 dpi, and increased further in the infected plants by 6 dpi (Fig. 6A). At both times, the plants infected with XccWT or Xcc∆otsAc contained about three times more trehalose than those infected with the XccΔotsA mutant. T6P levels were very similar in infected and mock-inoculated plants at 3 dpi, but were twice as high in the plants infected with XccWT or XccΔotsAc at 6 dpi compared with plants infected with the mutant XccΔotsA (Fig. 6B). Interestingly, another disaccharide monophosphate, α-M1P, was elevated in all of the infected plants, being highest in the plants infected with XccWT and XccΔotsAc (Fig. 6C) and thus showing a very similar behaviour to trehalose. This was confirmed by the high Pearson’s correlation coefficient (*r*) between trehalose and α-M1P contents at 3 dpi (*r*=0.882; *P*<0.05) and 6 dpi (*r*=0.730; *P*<0.05), whereas there was no significant correlation between trehalose and T6P (Supplementary Fig. S1 at *JXB* online).

A range of other sugar phosphates, glycolytic intermediates, and organic acids was also measured, but none of these showed significant differences between plants infected with the XccΔotsA mutant compared with the XccWT and XccΔotsAc controls (Supplementary Table S1 at *JXB* online). However, the infected plants had significantly lower levels of some glycolytic intermediates (glucose 6-phosphate, fructose 6-phosphate, fructose 1,6-bisphosphate, 3-phosphoglycerate, and phospho*enol*pyruvate) than the mock-inoculated controls, but higher levels of pyruvate, shikimate, and tricarboxylic acid cycle intermediates (citrate, aconitate, isocitrate, 2-oxoglutarate, succinate, fumarate, and malate) (Supplementary Table S1).

### Effect of Xcc infection on the proteome of *C. sinensis* leaves

Proteomic analysis of infected *C. sinensis* leaves identified 37 proteins that were differentially expressed (≥1.2-fold difference; *P*≤0.05) in plants infected with XccWT or XccΔotsA (Table 1). The majority of these were associated with one of the following biological processes: (i) respiration (glycolytic and tricarboxylic acid cycle enzymes, and mitochondrial ATP synthase); (ii) protein synthesis and turnover (ribosomal proteins, translation, protein folding, chaperonins, and targeted protein degradation); (iii) detoxification; and (iv) defence. Most of the differentially expressed proteins in these four categories were less abundant in plants infected with the XccΔotsA mutant than in those infected with XccWT. Two proteins of note were upregulated in XccΔotsA-infected plants: ribulose-bisphosphate carboxylase/oxygenase (RuBisCO) and a putative type 1 isopentenyl-diphosphate δ-isomerase (Table 1), which has a role in chlorophyll and carotenoid biosynthesis (Berthelot *et al.*, 2012). Thus, both the light-harvesting and CO_2_-fixing aspects of photosynthesis might be differentially affected by infection with the mutant compared with the WT strains of Xcc.

**Table 1.** Differentially citrus expressed protein spots between leaves infected with XccWT and Xcc∆otsA strains at 3 dpi and with a changed abundance of a minimum of 1.2-fold and P<0.05 (ANOVA) FunCat, Functional Catalogue; MW, molecular weight; p*I*, isoelectric point.

FunCat no./protein name	Accession no.	MOWSE score	Species	Predicted MW/pI	Observed MW/pI	Match/coverage	Fold change at 3 dpi (Xcc∆ostA vs XccWT)
01 Metabolism							
01.01 Amino acid metabolism							
Adenine phosphoribosyltransferase 1	APT1_WHEAT	25	*Triticum aestivum*	19.7/5.02	14/5.20	1/8%	1.4 +
*S*-Adenosylmethionine synthetase	Q1H960_ORYRU	77	*Oryza rufipogon*	42.6/5.74	40/5.20	3/6%	1.6 +
01.07 Metabolism of vitamins, cofactors and prosthetic groups							
NAD-dependent epimerase/dehydratase family protein-like protein	Q2XPW6_SOLTU	49	*Solanum tuberosum*	45.4/6.11	40/4.70	1/2%	1.2 –
01.20 Secondary metabolism							
Caffeic acid 3-*O*-methyltransferase	Q1JUZ5_IPONI	76	*Ipomoea nil*	39.9/5.71	32/5.00	3/6%	1.2 –
Isopentenyl-diphosphate delta-isomerase, type 1, putative	Q1S445_MEDTR	70	*Medicago truncatula*	27.5/5.07	19/5.70	2/5%	1.5 +
02 Energy							
02.01 Glycolysis and gluconeogenesis							
Fructose bisphosphate aldolase 1	AT2G21330	136	*Arabidopsis thaliana*	42.9/6.18	31/4.70	3/3%	1.3 –
Phosphoglycerate kinase	Q1SNT0_MEDTR	816	*Medicago truncatula*	50.0/6.64	34/5.10	28/10%	1.3 –
Apgm protein	Q9ZS53_MALDO	242	*Malus sylvestris*	60.8/5.44	58/4.90	7/6%	1.4 –
Malate dehydrogenase (oxaloacetate-decarboxylating) (NADP) 2	T06402	116	*Solanum lycopersicum*	64.1/5.71	60/4.70	8/4%	1.4 –
Triophosphate isomerase-like protein	Q3HRV9_SOLTU	143	*Solanum tuberosum*	27.7/5.90	17/4.80	8/14%	1.3 –
02.10 Tricarboxylic acid pathway							
Putative pyruvate dehydrogenase E1 β-subunit isoform 1 protein	Q6Z1G7_ORYSA	73	*Oryza sativa*	39.9/5.25	31/5.40	4/5%	1.4 –
Lipoamide dehydrogenase	Q6QJL7_CAPAN	170	*Capsicum annuum*	53.3/6.72	56/4.30	16/11%	2 –
Cytosolic aconitase	Q9FVE9_TOBAC	62	*Nicotiana tabacum*	98.0/5.88	70/4.30	4/3%	1.2 –
02.30 Photosynthesis							
Ribulose-bisphosphate carboxylases (RuBisCo)	ATCG00490	73	*Arabidopsis thaliana*	52.9/5.88	95/4.40	3/1%	1.4+
02.45 Energy conversion and regeneration							
Putative mitochondrial ATP synthase β-subunit	Q5N7P9_ORYSA	132	*Oryza sativa*	45.9/5.33	50/5.40	15/10%	1.4 –
Mitochondrial ATP synthase α/β family protein	AT5G08670	35	*Arabidopsis thaliana*	59.6/6.13	29/4.40	1/5%	1.2 –
12 Protein synthesis							
12.01 Ribosome biogenesis							
12.01.01 Ribosomal proteins							
Ribosomal protein L10	Q1SYJ3_MEDTR	70	*Medicago truncatula*	34.4/5.24	30/5.40	1/3%	1.6 +
Acidic ribosomal P2-like protein	PA0019	25	*Arabidopsis thaliana*	1.3/8.31	34/5.30	3/91%	1.2 –
12.04 Translation							
Translation initiation factor 4A-2	AT1G54270	61	*Arabidopsis thaliana*	46.7/5.45	34/4.90	2/4%	1.3 –
Partial elongation factor TuA	BAA01974	219	*Nicotiana sylvestris*	49.7/6.09	35/5.20	5/8%	1.3 –
14 Protein fate (folding, modification and destination)							
14.01 Protein folding and stabilization							
Peptidyl-prolyl *cis*-*trans* isomerase CYP38	CYP38_ARATH	57	*Arabidopsis thaliana*	48.0/5.06	35/6.30	4/7%	1.6 –
Heat-shock protein 70 family protein	AT3G09440	536	*Arabidopsis thaliana*	71.1/4.97	62/5.70	23/16%	1.6 +
Heat-shock protein 70	AT3G12580	394	*Arabidopsis thaliana*	71.1/5.14	62/5.80	23/15%	1.4 –
Hsp90-2-like	Q2XTE5_SOLTU	57	*Solanum tuberosum*	80.4/5.08	64/5.60	4/5%	1.7 –
14.13 Protein/peptide degradation							
Proteasome subunit α type-6	Q9XG77	48	*Nicotiana tabacum*	27.3/5.92	20/4.60	2/8%	1.3 –
AAA ATPase; 26S proteasome subunit P45	Q1RSM6_MEDTR	122	*Medicago truncatula*	47.4/4.98	45/5.90	7/12%	1.2 –
20S proteasome β-subunit D1	AT3G22630	34	*Arabidopsis thaliana*	22.5/5.95	14/4.80	3/5%	1.4 –
Proteasome subunit β type-7-B	PSB7B_ARATH	14	*Arabidopsis thaliana*	29.6/6.71	40/4.30	1/3%	1.3 –
30 Cellular communication/signal transduction mechanism							
Lectin-related protein	Q9FQ12_CITPA	62	*Citrus paradisi*	29.1/5.10	27/5.20	1/4%	1.6 –
Ras small GTPase, Rab type	Q1S416_MEDTR	44	*Medicago truncatula*	25.2/6.38	21/4.20	4/9%	1.6 –
Putative blue light receptor	Q8LPD9_CHLRE	35	*Chlamydomonas reinhardtii*	81.3/8.67	17/6.30	3/1%	1.5 +
32 Cell rescue, defence, and virulence							
32.05 Disease, virulence, and defence							
Latex protein allergen (Hev b 7)	Q9SEM0_HEVBR	67	*Hevea brasiliensis*	42.8/5.00	35/5.70	1/3%	1.3 –
32.07 Detoxification							
2-Cys peroxiredoxin BAS1 (PeroxBAS)	BAS1_HORVU	34	*Hordeum vulgare*	23.3/5.48	15/6.10	1/3%	1.4 –
Superoxide dismutase (SOD)	DSPMN	192	*Pisum sativum*	26.6/7.16	14/4.70	4/6%	1.2 –
Glutathione transferase lambda 2 (GST)	AT3G55040	131	*Arabidopsis thaliana*	33.0/6.78	19/5.50	10/3%	1.3 –
Lactoylglutathione lyase (glyoxalase I)	O04428_CITPA	295	*Citrus paradisi*	32.6/5.46	27/5.40	17/24%	1.5 –
NADP-dependent alkenal double-bond reductase P2	CAN61472	118	*Vitis vinifera*	40.4/5.94	30/5.80	4/2%	1.6 –

### Analyses of the photosynthetic efficiency in *C. sinensis* leaves infected with XccWT and the XccΔotsA mutant

**Fig. 7.**
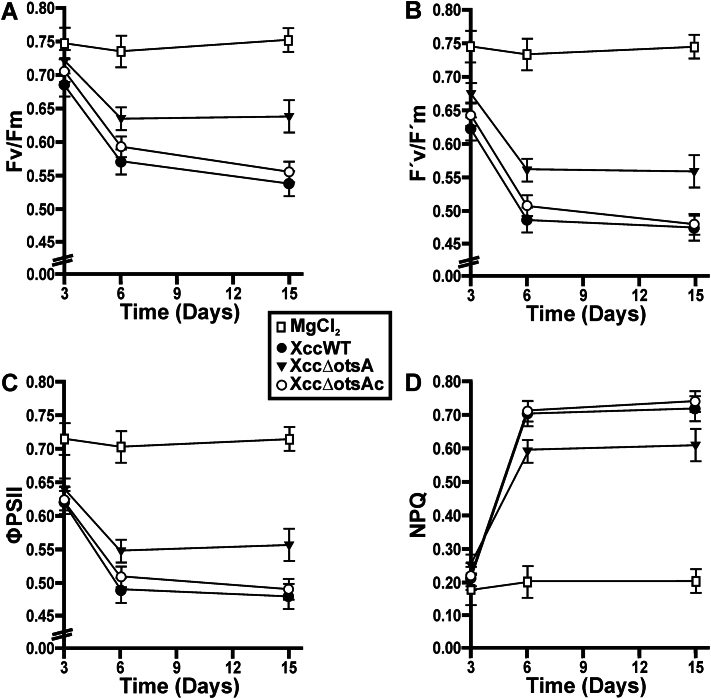
Photosynthetic parameters measurements in leaves infected with the XccWT, Xcc∆otsA, and Xcc∆otsAc strains. Quantification of the maximum quantum efficiency of PSII (Fv/Fm) (A), the effective quantum efficiency of PSII (F’v/F’m) (B), the PSII operating efficiency (ΦPSII) (C),and non-photochemical quenching (NPQ) (D) at different dpi in leaves infiltrated with the strains and with MgCl_2_ as a control. Values are the mean of five replicates and error bars represent the standard deviation. Data were statically analysed by one-way ANOVA (*P*<0.05).

Chlorophyll fluorescence parameters (Baker and Rosenqvist, 2004) were examined in leaves infiltrated with XccWT, Xcc∆otsA, or Xcc∆otsAc at 10^7^ cfu ml^–1^, or mock-infiltrated with 10mM MgCl_2_. Leaves infected with XccWT or Xcc∆otsAc strains showed reduced values of maximum quantum efficiency of photosystem II (PSII) (Fv/Fm) compared with MgCl_2_-infiltrated leaves at 3, 6, and 14 dpi (Fig. 7A). Infection with XccΔotsA also decreased Fv/Fm but to a significantly (*P*<0.05) lesser extent than infection with the other two strains (Fig. 7A). The same behaviour was observed for the effective quantum efficiency of PSII (F’v/F’m) (Fig. 7B) and the PSII operating efficiency (ΦPSII) (Fig. 7C). Non-photochemical quenching was higher in infected leaves, suggesting a greater loss of energy as heat compared with the non-infected controls (Fig. 7D). However, at 6 dpi, the increase in non-photochemical quenching was significantly (*P*<0.05) lower in Xcc∆otsA mutant-infected leaves than in those infected with XccWT or Xcc∆otsAc (Fig. 7D).

### Responses of *C. sinensis* leaves after Xcc infection

**Fig. 8.**
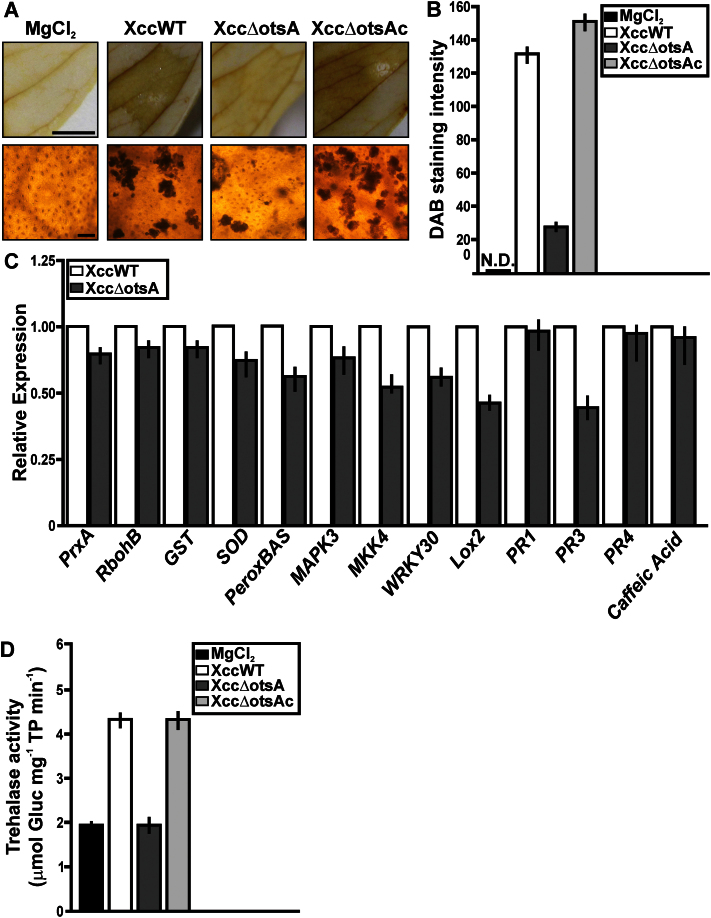
Analysis of ROS production, RT-qPCR of citrus genes related to defence responses, and trehalase activity in citrus leaves infected with the XccWT, Xcc∆otsA, and Xcc∆otsAc strains. (A) DAB detection of H_2_O_2_ accumulation in citrus leaves infected with the strains at 3 dpi. Representative photographs of DAB stained leaves are shown in the upper panels and microscopic observations in the lower panels. Bars, 10mm (upper panel); 1mm (lower panel). (B) Quantification of DAB staining from the microscopic imagines. The means were calculated from 25 photographs from three independent experiments for each strain. Error bars indicate standard deviation. N.D., not detected. (C) RT-qPCR of citrus genes related to defence responses. Bars indicate the expression levels of the genes from RNA extracted from leaves infected with the different strains and MgCl_2_-infiltrated leaves at 1 dpi. Values are the means of four biological replicates with three technical replicates each. Error bars indicate standard deviation. (D) Quantification of trehalase activity [μmol of glucose (Gluc) mg^–1^ of total protein (TP) min^–1^] in leaves infiltrated with the strains at 3 dpi. Bars are the means of five infected leaves. Error bars indicate standard deviation. Results were analysed by Student’s *t*-test (*P*<0.05) and one-way ANOVA (*P*<0.05).(This figure is available in colour at *JXB* online.)

The differential expression of several proteins involved in detoxification of reactive oxygen species (ROS) led us to investigate H_2_O_2_ production in citrus leaves infected with the various Xcc strains. At 3 dpi, the levels of H_2_O_2_, assessed by staining with DAB were significantly higher in XccWT- or Xcc∆otsAc-infected tissues than in those infected with the Xcc∆otsA mutant, and non-infected leaves did not show any staining (Fig. 8A, B). RT-qPCR analysis showed that transcripts of two diagnostic marker genes for oxidative stress (Garavaglia *et al.*, 2010; Sgro *et al.*, 2012), *SUPEROXIDE DISMUTASE* (*SOD*) and *2-CYS PEROXIREDOXIN BAS* (*PeroxBAS*), were significantly (*P*<0.05) less abundant at 1 dpi in plants infected with the XccΔotsA mutant than in plants infected with XccWT (Fig. 8C). Three other oxidative stress marker genes, *PEROXIREDOXIN* (*PrxA*), *NADPH OXIDASE* (*RbohB*), and *GLUTATHIONE-S-TRANSFERASE* (*GST*), showed a similar but not statistically significant trend. Similarly, several defence response-related genes were less expressed (*P*<0.05) in the XccΔotsA-infected plants, including: *MITOGEN ACTIVATED PROTEIN KINASE 3* (*MAPK3*), *MAP KINASE KINASE 4* (*MKK4*), *WRKY30* transcription factor, *LIPOXYGENASE 2* (*LOX2*), and *PATHOGENESIS RELATED 3* (*PR3*) (Fig. 8C). All of these genes showed the same levels of expression in plants infected with the complemented Xcc∆otsAc strain as those infected with XccWT (data not shown). At 3 dpi, plants infected with XccWT or XccΔotsAc had over twice as much trehalase activity compared with plants infected with XccΔotsA or non-infected plants (Fig. 8D).

### Oxidative stress and defence responses in *C. sinensis* leaves treated with exogenous trehalose

**Fig. 9.**
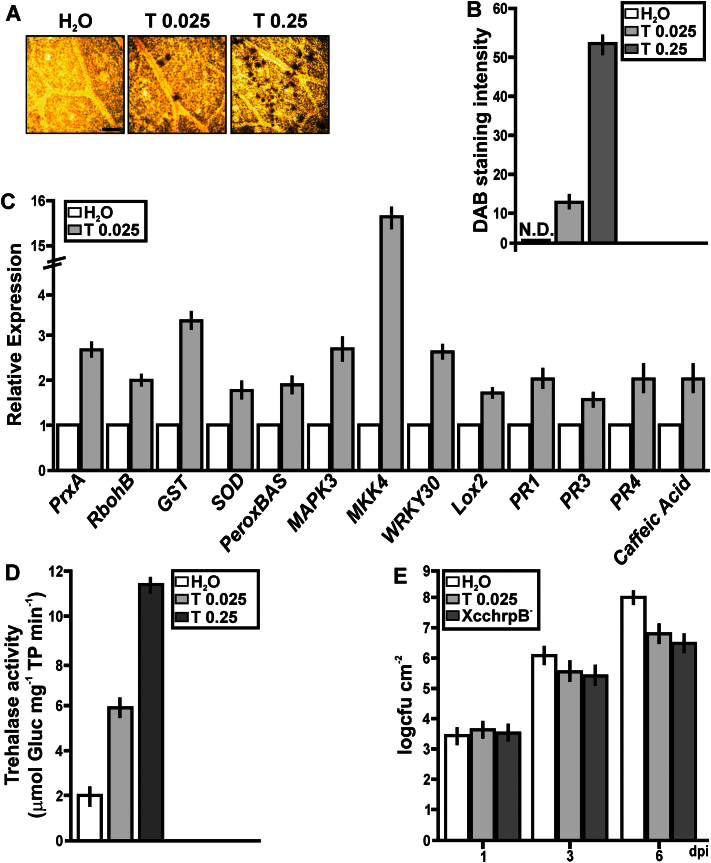
Analysis of ROS production, RT-qPCR of citrus genes related to defence responses, trehalase activity, and induction of the citrus response to XccWT in citrus leaves infiltrated with pure trehalose. (A) Representative microscopic photographs showing DAB staining of leaves infiltrated with 0.025 and 0.25mg ml^–1^ trehalose (T) and H_2_O as a control. Bar, 1mm. (B) Quantification of DAB staining from the microscopic images. Results are the means of 25 photographs from three independent experiments for each treatment. Error bars indicate standard deviation. N.D., not detected. (C) RT-qPCR of citrus genes related to defence responses. Results indicate the expression levels of the genes from RNA extracted from leaves infiltrated with 0.025mg ml^–1^ of trehalose at 1h post-infiltration relative to the expression levels found in H_2_O-infiltrated leaves. Values are the means of four biological replicates with three technical replicates each. Error bars indicate standard deviation. (D) Quantification of trehalase activity [μmol of glucose (Gluc) mg^–1^ of total protein (TP) min^–1^] in leaves infiltrated with 0.025 and 0.25mg ml^–1^ of trehalose or H_2_O as a control at 1h trehalose at 1h post-infiltration. Bars are the means of five infiltrated leaves. Error bars indicate standard deviation. (E) Quantification of XccWT growth in citrus leaves pre-infiltrated with 0.025mg ml^–1^ of trehalose, or with H_2_O or the XcchrpB^–^ strain as controls. Values are the means obtained from 10 infiltrated citrus leaves at different dpi. Error bars show the standard deviation. Results were analysed by Student’s *t*-test (*P*<0.05) and one-way ANOVA (*P*<0.05). (This figure is available in colour at *JXB* online.)

*C. sinensis* leaves were infiltrated with trehalose (0.025 or 0.25mg ml^–1^) or water (control) to investigate whether the more pronounced oxidative stress and defence responses in the XccWT-infected plants were directly attributable to the higher trehalose content of XccWT. Even at the highest concentration of trehalose used (0.25mg ml^–1^=0.73mM), the osmotic potential of the infiltration solution was be well below that of the cytoplasm and was therefore unlikely to cause osmotic stress. Light microscopic analysis of the leaves confirmed that the mesophyll cells were not plasmolysed and showed no other evidence of cell damage from the infiltration procedure (data not shown). Control leaves showed little or no staining with DAB, whereas leaves infiltrated with trehalose showed a strong reaction, increasing with the concentration of trehalose (Fig. 9A, B). All of the marker genes for oxidative stress or defence responses that were differentially expressed in XccΔotsA- versus XccWT-infected plants were also significantly induced by trehalose (Fig. 9C). In addition, some other defence-related genes (*PR1*, *PR4*, and *CAFFEIC ACID 3-O-METHYLTRANSFERASE*) were significantly induced by trehalose (Fig. 9C), even though they were not differentially expressed in XccWT- and XccΔotsA-infected plants (Fig. 8C). Within 1h of trehalose infiltration, trehalase activity was three times higher in leaves treated with 0.025mg ml^–1^ of trehalose than in the water control, and almost six times higher after treatment with 0.25mg ml^–1^ of trehalose (Fig. 9D). These results indicated that trehalose can promote defence responses in citrus leaves. Therefore, the potential effect of this sugar to enhance canker disease resistance was investigated. Citrus leaves were pre-treated with 0.025mg ml^–1^ of trehalose or water as a control and also with the XcchrpB^–^ mutant strain, which does not produce infection symptoms (Zimaro *et al.*, 2014) but enhances citrus defence responses (data not shown). The pre-treated tissues were then infiltrated with XccWT at 10^6^ cfu ml^–1^ and bacterial growth was monitored up to 6 dpi. At 6 dpi, the population density of XccWT was significantly lower (*P*<0.05) in the trehalose- and XcchrpB^–^-treated leaves, being about 10 times less than in the water-treated control (Fig. 9E). RT-qPCR analysis of the marker genes for oxidative stress or defence responses was performed in *C. sinensis* leaves infiltrated with the disaccharides maltose and sucrose at 0.025mg ml^–1^. Maltose did not induce the expression of any of these genes compared with the water control (*P*<0.05) (data not shown). However, the expression of *PrxA*, *MAPK3*, *MKK4*, and *WRKY30* was significantly induced by sucrose but to a lesser extent than with trehalose (Supplementary Fig. S2 at *JXB* online). Defence gene induction with sucrose, a recognized plant defence signal in response to pathogens (Moghaddam and Van den Ende, 2012), did occur; however, the citrus defence responses were more pronounced in the presence of trehalose than sucrose, suggesting that plant responses to trehalose are different and are independent of sucrose responses.

## Discussion

There are currently five known pathways for trehalose biosynthesis described in the three domains of the tree of life (Iturriaga *et al.*, 2009). The Xcc genome (da Silva *et al.*, 2002) contains genes encoding enzymes of three of these biosynthetic pathways: the otsA–otsB (TPS–TPP), glgY–glgZ, and treS pathways. Gene expression analysis (Fig. 1) indicated that the otsA–otsB pathway is the main route for trehalose biosynthesis in cells grown in XVM2 medium, confirming a previous observation (Astua-Monge *et al.*, 2005), and that this pathway is induced during plant infection. Expression of *otsB* was also upregulated in a different pathovar, *X. campestris* pv. *campestris*, when grown in a medium used in pathogenicity studies (Liu *et al.*, 2013). These findings suggest that trehalose biosynthesis via the otsA–otsB pathway is functionally significant in *Xanthomonas*–plant interactions. To understand better the role of this pathway in Xcc pathogenicity, we generated an *otsA* deletion mutant of Xcc, which lacked the capacity to synthesize trehalose via this route, and this resulted in a lower trehalose content when grown in XVM2 medium (Fig. 2A). This mutant still produced trehalose, as the glgY–glgZ and treS pathways may be active under these growth conditions; however, the lower levels of trehalose due to the absence of the otsA–otsB pathways have detrimental effects on bacteria. In comparison with the WT and complemented strains, the XccΔotsA mutant: (i) grew less well in culture when exposed to salt or oxidative stress (Fig. 2B); (ii) survived less well on the surface of *C. sinensis* leaves (Fig. 3A); and (iii) did not cause canker lesions in leaves infected using a natural method of infection (Fig. 3B). This indicated that trehalose synthesized via the otsA–otsB pathway helps to maintain cell viability during the pre-infection phase, when the bacteria are exposed to high light, UV, and water stress in the inhospitable environment of the leaf surface. This finding corroborates a previous study showing that trehalose is important for the survival and fitness of *Pseudomonas syringae* in the phylloplane (Freeman *et al.*, 2010).

When the Xcc strains were infiltrated directly into leaves, eliminating any differences in the pre-infection phase, at lower bacterial concentrations the Xcc∆otsA mutant was less able to infect the plant cells and caused fewer canker lesions (Fig. 4). In leaves infiltrated with higher bacterial concentrations, the Xcc∆ostA mutant produced lesions with fewer and smaller necrotic areas, while bacterial growth was similar for all the strains (Fig.4). The presence of larger necrotic areas in infections with XccWT may be due to the fact that trehalose induces defence responses in citrus leaves, enhancing bacterial-induced tissue necrosis. The similarity in growth observed for both XccWT and the Xcc∆ostA mutant under the latter infection conditions may be related to the presence of a larger amount of healthy tissue at the mutant infection site, and this may enhance the survival of the mutant. Accordingly, we previously described that citrus tissue necrosis impairs Xcc growth (Gottig *et al.*, 2008). The co-infiltration of exogenous trehalose with lower bacterial concentrations impairs canker formation, suggesting that, for bacteria, it is more difficult to counteract plant defence responses when they are at a lower population density (Fig. 4). Leaf tissue infected with XccΔotsA contained less trehalose than tissue infected with the WT or complemented strain but more than mock-inoculated leaves (Fig. 6A). Most of the trehalose in the XccΔotsA-infected tissue is likely to be derived from the bacteria themselves, as these still contain trehalose despite the loss of the otsA–tsB pathway (Fig. 2A). However, a contribution from the plant is also a possibility. Conceptually, infection with Xcc might increase the level of trehalose by inducing the plant’s own trehalose biosynthetic pathway or repressing its trehalase activity. However, we can exclude the latter possibility, as trehalase activity was not affected in plants infected with XccΔotsA, and actually increased in plants infected with the WT or complemented strain (Fig. 8D).

The T6P detected in XccΔotsA-infected tissues (Fig. 6B) must have come from the plant cells as the bacteria lack the capacity to synthesize this intermediate. At 3 dpi, similar T6P levels were observed in mock-inoculated leaves, where the plant was also the only possible source, as well as in leaves infected with the WT or complemented strain (Fig. 6B). This suggests that either the XccWT and XccΔotsAc cells contributed little of the T6P detected in the infected tissue, or that any contribution from the bacterial cells was exactly matched by a decrease in plant-derived T6P, which seems unlikely. However, at 6 dpi, there was twice as much T6P in the XccWT- and XccΔotsAc-infected leaves, which might reflect the decreased expression of *otsB* (Fig. 1C), leading to accumulation of T6P in the bacterial cells. Another possibility is that the increase reflects a rise in T6P levels in the plant cells in response to successful infection by the virulent WT and complemented strains. Consumption of apoplastic sucrose by the bacteria as they proliferate throughout the plant tissue is likely to perturb sucrose levels within the cells, and this in turn would be expected to affect T6P, which acts as a signal of sucrose status in plant cells (Lunn *et al.*, 2006; Yadav *et al.*, 2014).

The differences in trehalose content between mock-inoculated and Xcc-infected leaves were almost exactly mirrored by those in α-M1P (Fig. 6 and Supplementary Fig. S1C, D). Spinach chloroplasts were reported to contain an unspecified isomer of maltose phosphate (Schilling *et al.*, 1976), but LC-MS/MS analysis of axenic *Arabidopsis thaliana* seedlings found no evidence of α-M1P (R. Feil and J.E. Lunn, unpublished data). In *C. sinensis*, no α-M1P was detected in non-infected leaves. As α-M1P is an intermediate in a recently described bacterial pathway of glucan synthesis from trehalose (Miah *et al.*, 2013), it seems likely that the α-M1P in infected leaves had a bacterial origin. In the bacterial glucan synthesis pathway, trehalose is reversibly converted by treS to maltose, which is then phosphorylated to α-M1P by maltokinase (Drepper *et al.*, 1996). In Xcc, these two activities reside in the same bifunctional enzyme encoded by XAC0155, with an N-terminal treS domain and C-terminal maltokinase domain. The resulting α-M1P is the substrate for synthesis of linear α-1,4-glucan chains by glucan synthase (glgE; XAC0154), which are then branched by a branching enzyme (glgB; XAC0156) to form an α-1,6-branched α-1,4 glucan similar to glycogen, which is used as a storage carbohydrate. The presence of genes encoding all the necessary enzymes in the Xcc genome, and the detection of the diagnostic intermediate, α-M1P, in Xcc-infected leaves, which otherwise lack the metabolite, shows that the pathway is likely to be operating in Xcc cells. To date, the role of this pathway in plant-pathogenic bacteria has not been elucidated.

The proteomic analysis performed with Xcc-infiltrated leaves suggests that pathogen trehalose modulates enzymes involved in host carbon metabolism including photosynthesis (Table 1). Trehalose produced by *P. aeruginosa* strain PA14 is a strong virulence factor necessary for the acquisition of nitrogen-containing nutrients, and thus for pathogen growth in the intercellular environment of *Arabidopsis thaliana* leaves (Djonovic *et al.*, 2013). Likewise, a study of the interaction of *Arabidopsis thaliana* with *Plasmodiophora brassicae* suggested that trehalose released into the plant by the pathogen is able to exploit the plant’s trehalose-sensing system and alter carbohydrate metabolism to the benefit of the pathogen (Brodmann *et al.*, 2002). In legumes, trehalose produced by symbiotic microorganisms induces sucrose synthase and alkaline invertase activities, suggesting that trehalose could have a role in providing sugars to the rhizobial symbiont (Müller *et al.*, 1998). Moreover, in ectomycorrhizal symbiosis, it has been suggested that trehalose could create a carbon sink for the symbiont, thereby attracting photoassimilates (Lopez *et al.*, 2007).

Through gene expression and proteomic analyses, we showed that several antioxidant enzymes were downregulated in tissues infected with Xcc∆otsA compared with XccWT (Fig. 8C, Table 1). This was supported by the results of the DAB staining, which showed that XccWT triggered a larger accumulation of H_2_O_2_ than the Xcc∆otsA mutant (Fig. 8A, B). Furthermore, several defence-related genes were expressed at a lower level in citrus leaves infected with the Xcc∆otsA mutant relative to XccWT (Fig. 8C). The differences at the transcript level were also reflected in the differential abundance of several proteins related to plant defence responses, which were also downregulated in leaves infected with the Xcc∆otsA mutant compared with XccWT (Table 1). To our knowledge, this is the first time that a differential plant defence response towards a pathogen impaired in trehalose production was observed.

In addition, we analysed citrus responses to the presence of exogenous trehalose. The expression levels of genes that encode antioxidant enzymes and also ROS accumulation were induced in citrus leaves treated with pure trehalose (Fig. 9C). Some previous studies have shown that several genes of *Arabidopsis thaliana* that respond to exogenous trehalose treatments are related to ROS and secondary metabolism activation, suggesting that trehalose triggers an induction of ROS signalling (Bae *et al.*, 2005a; Aghdasi *et al.*, 2008). Furthermore, a transcriptome analysis revealed that potato transgenic lines that overproduce T6P have higher levels of gene transcripts that encode antioxidant enzymes (Kondrak *et al.*, 2011). Moreover, infiltration of trehalose by itself was sufficient to induce expression of citrus defence genes (Fig. 9C), supporting a role for trehalose as an enhancer of defence responses in *Citrus* spp. Accordingly, some genes that were induced in response to trehalose in *Arabidopsis thaliana* seedlings exposed to this disaccharide are known to be involved in biotic interactions, providing yet further evidence that trehalose triggers plant defence responses (Aghdasi *et al.*, 2008). In addition, we observed that the pre-infiltration of citrus leaves with this disaccharide impaired Xcc growth after inoculation (Fig. 9E). In a similar study, it was shown that trehalose application on wheat plants induced resistance to *Blumeria graminis* (Reignault *et al.*, 2001; Renard-Merlier *et al.*, 2007; Tayeh *et al.*, 2014).

Citrus trehalase might also serve to counteract the detrimental effects of excess trehalose derived from the Xcc pathogen, since trehalase activity was higher in leaves infected with the virulent XccWT strain than the non-virulent Xcc∆otsA (Fig. 8C). In a previous report of *Plasmodiophora brassicae–Arabidopsis thaliana* interaction, it has been proposed that trehalose is secreted by the pathogen and that *Arabidopsis thaliana* trehalase (AtTRE1) may sense trehalose, and induction of trehalase activity might serve as a defence against excessive accumulation of trehalose (Brodmann *et al.*, 2002).

This study revealed a dual role for trehalose and the bacterial otsA–otsB pathway during infection of *Citrus* spp. by Xcc. Before infection, trehalose protects the bacterial cells from oxidative and water stress while they are on the leaf surface and exposed to direct sunlight. While trehalose could be important for manipulating host carbohydrate metabolism in favour of the bacteria, perhaps by perturbing the plant’s endogenous trehalose metabolism and T6P levels, there is also evidence that the plant senses extracellular trehalose from the bacteria as a signal of pathogen attack, triggering defence responses to counteract infection by the pathogen. Thus, trehalose is a double-edged sword for both partners in the citrus–Xcc interaction, and our results add weight to the evidence that trehalose is a key factor in the never-ending struggle between plant pathogens and their host plants.

## Supplementary data

Supplementary data are available at JXB online.

Supplementary Fig. S1. Relationships between trehalose, T6P and α-Mal1P in leaves infected with XccWT.

Supplementary Fig. S2. RT-qPCR of citrus genes related to defence responses in citrus leaves infiltrated with pure trehalose and sucrose.

Supplementary Table S1. Quantification of metabolite levels in infected citrus leaves.

Supplementary Table S2. Oligonucleotides used in qRT-PCR assays.

Supplementary Data

## Abbreviations:

ANOVA: analysis of variance
cfu: colony-forming units
DAB: 3,3′-diaminobenzidine
dpi: d post-inoculation
EPS: exopolysaccharide
LC-MS/MS: liquid chromatography coupled to tandem mass spectrometry
RT-qPCR: quantitative reverse transcription-PCR
α-M1P: α-maltose 1-phosphate
PSII: photosystem II
ROS: reactive oxygen species
T6P: trehalose-6-phosphate
TPS: T6P synthase
T3SS: type III secretion system
WT: wild type
Xcc: *Xanthomonas citri* subsp. *citri* .
