# Supplementary material for: The dual nature of trehalose in citrus canker disease: a virulence factor for Xanthomonas citri subsp. citri and a trigger for plant defence responses
Source: J Exp Bot. 2015 Mar 14;66(9):2795–811. doi: 10.1093/jxb/erv095 (PMC4986880; doi:10.1093/jxb/erv095)
Supplement: Supplementary Data [file supp_66_9_2795__index.html]

The dual nature of trehalose in citrus canker disease: a virulence factor for Xanthomonas citri subsp. citri and a trigger for plant defence responses — The dual nature of trehalose in citrus canker disease: a virulence factor for Xanthomonas citri subsp. citri and a trigger for plant defence responses — Supplementary Data 

# The dual nature of trehalose in citrus canker disease: a virulence factor for *Xanthomonas citri* subsp. *citri* and a trigger for plant defence responses

## Supplementary Data

Data files

**Files in this Data Supplement:**

- Supplementary Data - Supplementary Data
