# Supplementary material for: The dual nature of trehalose in citrus canker disease: a virulence factor for Xanthomonas citri subsp. citri and a trigger for plant defence responses
Source: J Exp Bot. 2015 Mar 14;66(9):2795–811. doi: 10.1093/jxb/erv095 (PMC4986880; doi:10.1093/jxb/erv095)
Supplement: Supplementary Data [file supp_erv095_jexbot141788_file001.pdf]

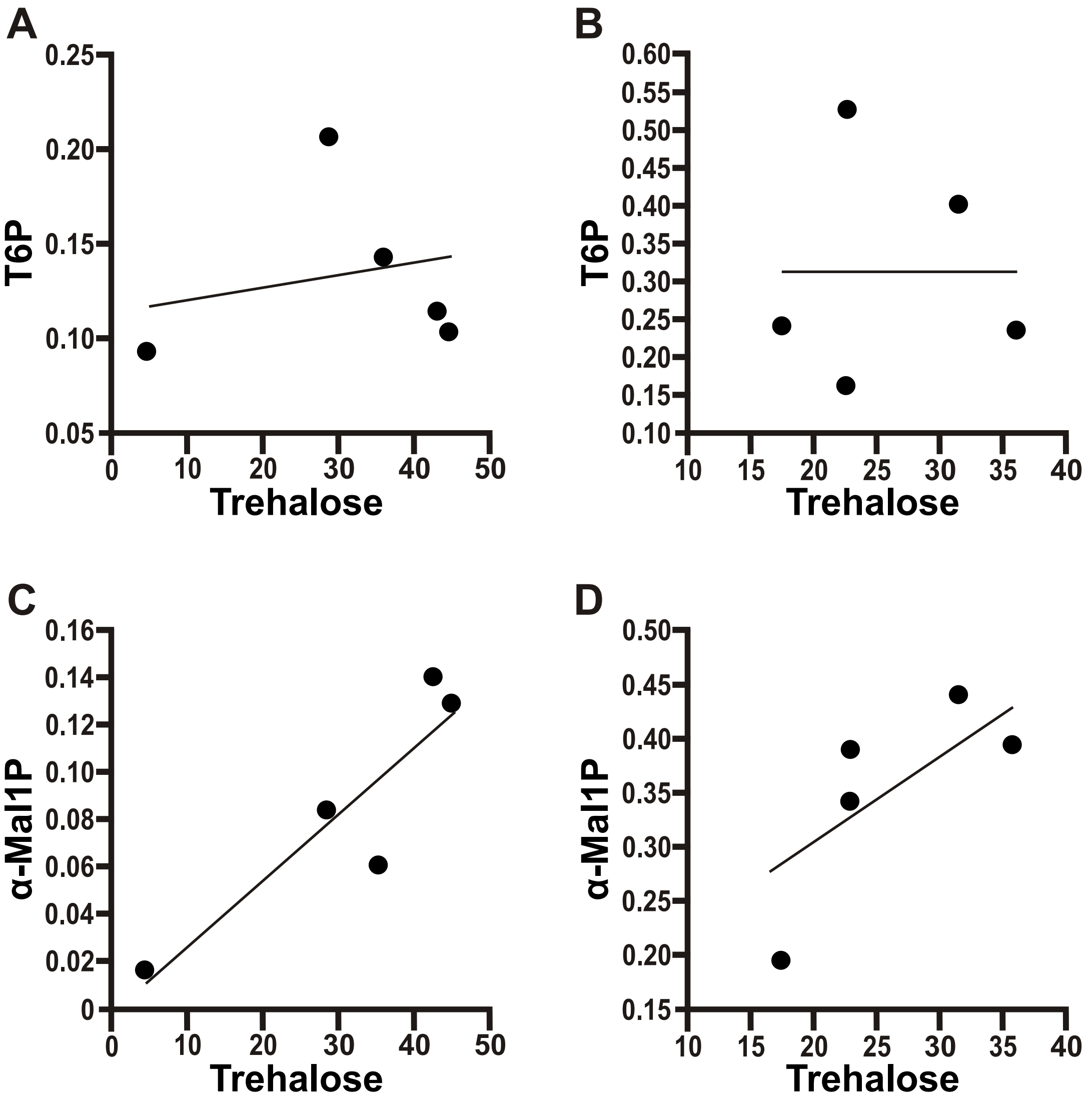

Supplementary Figure 1. Relationships between trehalose, T6P and -Mal1P in leaves infected with XccWT. The plots show pairwise comparisons of metabolite data from individual samples of leaves infected with XccWT at 3 dpi (**A** and **C**) and at 6 dpi (**B** and **D**). Results were analyzed by Student t-test ($p < 0.05$) and one-way ANOVA ($p < 0.05$).

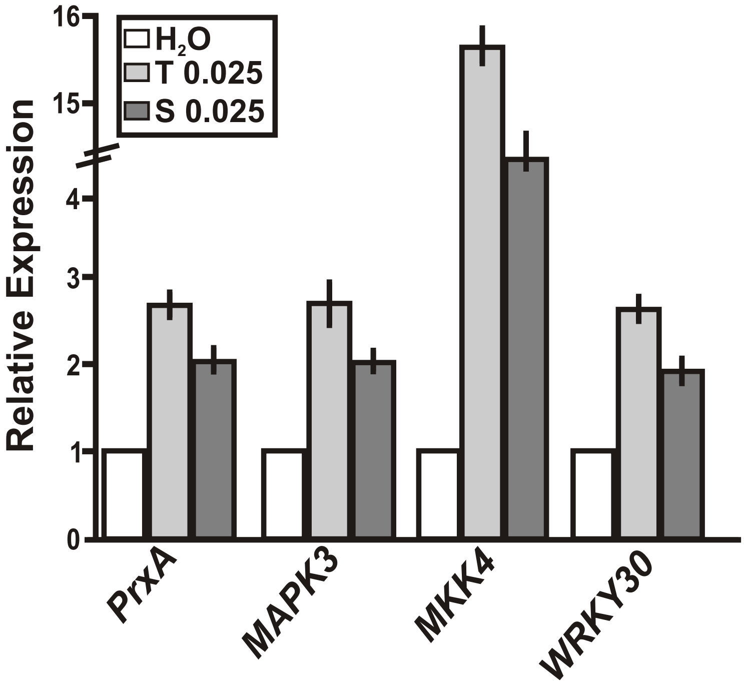

Supplementary Figure 2. RT-qPCR of citrus genes related with defence responses in citrus leaves infiltrated with pure trehalose and sucrose. RT-qPCR of citrus genes related with defence responses. Bars indicate the expression levels of the genes from RNA extracted from leaves infiltrated with 0.025 mg ml^{-1} trehalose (T) and sucrose (S) 1 hour post infiltration (hpi) relative to the expression levels found in H_2O-infiltrated leaves. Values are the means of four biological replicates with three technical replicates each. Error bars indicate standard deviations. Results were analyzed by Student t-test ($p < 0.05$) and one-way ANOVA ($p < 0.05$).

Supplementary Table 1. Quantification of metabolite levels in *citrus* leaves infected with XccWT and XccΔotsA strains at 3 dpi and 6 dpi. Results were analyzed by one-way ANOVA ($p < 0.05$).

	**Mean (nmol/g FW) ± SEM at 3 dpi**			**Mean (nmol/g FW) ± SEM at 6 dpi**		
	**$MgCl_2$**	**XccWT**	**XccΔostA**	**$MgCl_2$**	**XccWT**	**XccΔostA**
Citrate	2072.09±339.15	5754.02±650.78	5302.16±950.09	9089.54±973.61	10063.95±1359.35	13999.49±1242.33
Aconitate	16.22±1.64	51.02±2.12	51.96±3.58	28.41±3.29	72.78±6.14	127.76±11.78
2-Oxoglutarate	309.51±23.69	1425.31±102.90	2191.61±234.90	1053.21±173.95	1357.17±228.99	2536.69±328.43
Iso-Citrate	23.86±5.67	44.89±5.55	47.13±4.99	62.92±7.26	74.93±9.55	79.67±12.03
Succinate	151.11±32.61	464.42±19.66	400.34±23.32	100.65±32.61	402.88±13.64	423.93±36.69
Fumarate	132.53±9.44	222.56±13.70	186.52±10.09	163.78±10.15	337.93±22.68	255.16±11.94
Malate	10729.09±830.95	26607.63±1619.60	24402.70±2227.83	28584.83±3594.96	34026.73±3144.32	41918.73±3725.12
Piruvate	225.53±15.95	513.85±34.36	537.26±27.33	258.33±24.64	468.75±51.76	376.89±32.82
Phosphoenolpyruvate	6.67±0.39	0.82±0.34	1.42±0.52	10.04±1.82	0.15±0.07	0.53±0.16
Skikimate	54.43±6.65	252.39±33.41	163.22±18.69	54.43±6.65 [a]	72.56±12.20 [a]	50.96±9.57 [a]
Glycerate	698.49±122.00 [a]	728.27±88.13 [a]	891.04±119.37 [a]	725.00±90.86	253.79±39.37	433.00±43.43
3-Phosphoglycerate	39.21±4.31	9.13±2.28	12.59±1.67	36.85±5.94	0.92±0.25	3.19±0.64
Glycerol-3-phosphate	21.66±2.22	11.21±1.03	12.65±1.10	17.21±1.71	4.32±0.56	6.76±1.06
Sacarose-6-phosphate	0.28±0.01 [a]	0.31±0.02 [a]	0.28±0.02 [a]	0.42±0.05 [a]	0.35±0.05 [a]	0.25±0.02 [a]
Galactose-6-phosphate	0.87±0.08 [a]	0.49±0.18 [a]	0.68±0.13 [a]	1.76±0.22 [a]	0.70±0.35 [a]	0.20±0.08 [a]
Fructose-1,6-bisphosphate	2.47±0.38	1.07±0.37	1.94±0.48	0.85±0.24	0.077±0.0039	0.02±0.01
Fructose-6-phosphate	14.98±1.40 [a]	12.18±1.69 [a]	16.50±1.66 [a]	32.83±3.38	9.41±0.92	12.98±1.23
Glucose-6-phosphate	36.28±2.22 [a]	15.10±1.91 [a]	26.11±3.22 [a]	73.99±7.55	15.10±1.91	26.11±3.21
Glucose-1-phosphate	3.20±0.30	6.87±0.63	6.53±0.25	7.90±1.01 [a]	15.49±1.47 [a]	12.09±1.31 [a]
Maltose-6- phosphate	12.23±1.12 [a]	7.74±1.04 [a]	10.79±0.71 [a]	17.82±1.48	6.75±0.79	9.64±1.29
UDP-Glucose	22.34±6.59	50.56±4.75	71.64±4.69	58.88±4.83 [a]	61.99±4.13 [a]	73.18±4.19 [a]
ADP-Glucose	0.18±0.03	0.42±0.03	0.47±0.03	0.12±0.01	0.35±0.05	0.38±0.03

a Indicates that this metabolite did not change significantly between $MgCl_2$ treated and infected leaves.

Supplementary Table 2. Xcc and *C. sinensis* (Cs) oligonucleotides used in qRT-PCR assays.

Gene name	Forward Oligonucleotide	Reverse Oligonucleotide
otsA	ATGAGTCGTTTGGTGGTGG	GCTTGTTGAGGTCCATGGTG
otsB	TACCGTCGCCGCCATTGCTG	GCGAACAGGGCATCCAGCT
glgY	ATGATCGACCTCCGCGCTAC	TCGCCGCCGAGTTCGGGATTG
treS	ATGAATGCAGTGGCATCGTTAC	CAGCAGCCAGATGGTGTC
hrpG	ACAACATTCTGGCCTGGTAT	TTGTAGATGTGCTGCTCCAT
hrpX	CGATGATGAGGTCAGTTTGT	ACTGCGCAAAGCAATTCAAC
hrpB2	AACCAAGCGCTTGTGAATCG	CTATTGGTTCTTGACCAGTG
gumD	GCGCGGCCGTGGGATTGCTGAGT	TGGCGGCGCTGACGGAAGAACAC
16S	TGGTAGTCCACGCCCTAAACG	CTGGAAAGTTCCGTGGATGTC
CsLOB1	TCCACCAACCGAACCATACA	GGCACTTGCTTCATAGACCAT
CsPrxA	AGCCGCTCTCATTTCCTCTA	TTGATCGAAAACAGCCTCTG
CsRbohB	TCTAGGAAAATCAGGATTGTTATGTC	AAAGCCAGATAGATTCAGATACAAGA
CsGST	GTTCATCAGATATCTTAAGGCTGGTA	AACCTACTTGGAAACACACTAGAAGA
CsSOD	CAGAAGCATCACCAGGCTTA	CAATGCTTCCAGAGAACCAA
CsPeroxBas	ATTTTGCTGCAGAGGCTGTT	TGAAAACACACTGTCGGTTGA
CsMAPK3	TTACATGATGAAGCCGATGAAC	TGAGTGCTAATGCCTCCTGATA
CsMKK4	GGCACCCTCGATACTTTGTT	TAATTCCCTCCGTAGGCATC
CsWORKY30	CGAAAGAAGCAAATGGGGTA	GGAAATAAACGTGGCGAAAA
CsLox2	GTATAAATATTGCCCAAAGTTCACAG	GCCTTAAAACAATGGGTTACTAACTA
CsPR1	ACATGATCAATAGTAGGGATGTTAGC	AAAGTTGTTCAAACTTTTTGTCCTT
CsPR3	ATAAGAGCGTAACAGTTTAGTTGTCC	ACGGTCTAACCACAAATATAATCAAC
CsPR4	GTGTGATTCTGTCACTTTGTCTACTG	ACTGTTTGTGACCCTTAAGCAC
CsCaffeicAcid	AACCAGTGCCTCAGTCTTGC	CTGAATAGCTCGCCAGAAGG
CsActin	TCAATTGGATACTTCAAAGTCAAAAT	ACGTGAATTCTAGTGTTTCGATAAGT
